# Dr. Mercier and Sir Oliver Lodge

**Published:** 1917-07-21

**Authors:** 


### ON SPIRITUALISM.

### DR. MERCIER AND SIR OLIVER LODGE.

Dr. Mercier's literary exercises are always a source of satisfaction to his readers. Even opponents who suffer from his controversial dexterity cannot but admire the art with which the thrust is delivered. The rapier play is as graceful as it is effective, and the contest to the spectator is pure enjoyment. But not less is the combatant in earnest. The challenge of the lists has a serious purpose and seeks the triumph of a cause, not merely admiration of the tilting. It is this combination of expert skill and lofty motive which raises controversy to a high plane and makes it both an attractive and a helpful agent in the pursuit of truth. In a word, Dr. Mercier in a very special degree combines sincerity of purpose with clear thinking and apt speech, and hence his meaning is never doubtful, whether we elect for conversion or, alternatively, resolve to remain in our sins.

These characteristics of the author's method and style find a high level of expression in his latest volume.* The subject, of course, has its ludicrous aspect, and presents an obvious opportunity for ridicule. When we are informed that a plain, homely table, under the sway of a supernormal influence, "made most caressing movements," "seemed to be delighted with its new trick," and "shook as if laughing," we may be excused some sense of the ludicrous. And the reporter, even though he be the Principal of a university, must not be surprised if his reputation for common sense is pungently questioned. So much on the lighter side of Dr. Mercier's book. But, equally, the subject has its serious side, and the main part of the volume is occupied by serious argument. The pursuit of the occult has in recent days attracted many disciples. Its exponents occupy high places, and their published volumes are sold in thousands. Not faith but exact observation is claimed as its foundation, and the benediction of science is professed as its guarantee.

Apart from those who have embraced the cult there are many who have heard its appeal and are more or less shaken by it. Hence it becomes a matter of importance to test the validity of its methods and to examine the evidence which is offered as its justification. For, if its doctrines are true, there is settled a question which has agitated mankind since the days of the Witch of Endor, and this to-day, and with pathetic earnestness, many stricken hearts are asking. The issue, be it repeated, is placed on a plane where the strict rules of evidence are professed and the affirmative is offered as an inevitable outcome from the application of these rules. Dr. Mercier has accepted the challenge, and the present volume is the outcome of his decision.

Whether it will secure converts or even readers in the congregations of the elect may be doubted. But it will, we hope, steady public opinion; and the aim is a worthy one. Medical practitioners have special opportunities of knowing the extent to which the doctrines of spiritualism and telepathy are at present disturbing men and women in various orders of society, and the disturbance in certain individuals produces serious consequences. Hence we commend Dr. Mercier's book to our medical readers, not only for their personal entertainment, but also as an effective agent for the prevention of mischief in minds ready to be shaken by high-sounding professions. One further general remark may be added. The message of the modern spiritualists, at least in its substance, coincides with one of the messages delivered throughout the ages by the Christian Church. This latter rests upon its own grounds and has its own appeal. Neither the one nor the other is concerned in a discussion on the methods and teaching of spiritualism. The question at issue here is not whether, in view of its source and origin, belief in a future life is justified as an act of faith, but whether the proposition has been actually demonstrated by events which claim verification in accordance with the strictest conditions of scientific inquiry. To attack this profession of modern spiritualism has thus no impact on the position of those who rest in quiet assurance on teachings which advance an entirely different sanction. Whether these teachings are or are not justified is a debate which is not touched by an examination of the merits of telepathy or by a scrutiny of the evidence on which Sir Oliver Lodge rests his claim to have received messages from the dead. The matter here is one not of faith, but of the value of the testimony advanced in a particular field of observation, and it is to such an investigation that Dr. Mercier limits himself.

As we have already hinted, the purpose of the book is by no means to make sport for the Philistines. The conclusions are not wanting in severity, and irony, ridicule, and banter have their place in the argument. But the appeal is for an examination of the professed evidence by scientific method, and it is on the necessity of this method that Dr. Mercier is most anxious to insist. He, at least, has no doubt that if the method is applied his conclusions must follow, but here each reader is free to judge for himself. Personally we entirely agree with him, and we are unable to see any difference between the "discarnate intelligences" of to-day and the "ghosts" and "spooks" of a less polite generation, or how the professors of modern spiritualism can credit their mediums and yet refuse a faith in witchcraft. Distinguished men, however, maintain this position, and here is one of the attractions for the multitude. All the more reason why the question should be examined by one skilled in the weighing of evidence and able to show its real range and merit in such a fashion that even "wayfaring men shall not err therein." The results of this investigation are to be found in the later chapters of Dr. Mercier's book. The

* *Spiritualism and Sir Oliver Lodge.* By Charles A. Mercier, M.D. London: The Mental Culture Enterprise, 1917. Pp. 132 + xvi. Price 4s. 6d. net.

earlier chapters are occupied by a preliminary education of the reader in the nature of evidence and its interpretation. If the lessons here taught were universally practised the world might be a less entertaining place, but we should escape much unnecessary controversy and much contradiction of spirit. Dr. Mercier has written many books, and some of them, we fancy, have but a limited constituency. In his latest volume he speaks on a matter of high interest to many readers, and we hope he will receive the welcome due to a safe, competent, and entertaining guide.

